# Viral and bacterial investigations on the aetiology of recurrent pig neonatal diarrhoea cases in Spain

**DOI:** 10.1186/s40813-018-0083-8

**Published:** 2018-04-05

**Authors:** Susana Mesonero-Escuredo, Katrin Strutzberg-Minder, Carlos Casanovas, Joaquim Segalés

**Affiliations:** 1 IDT Biologika SL, Gran Vía Carles III, 84, 3°, 08028 Barcelona, Spain; 2 IVD Innovative Veterinary Diagnostics (IVD GmbH) Albert-Einstein-Str. 5, 30926 Seelze, Germany; 3 Departament de Sanitat i Anatomia Animals, Universitat Autònoma de Barcelona (UAB), 08193 Bellaterra, Barcelona, Spain; 4 UAB, Centre de Recerca en Sanitat Animal (CReSA, IRTA-UAB), Campus de la Universitat Autònoma de Barcelona, 08193 Bellaterra, Barcelona, Spain

**Keywords:** Neonatal diarrhoea, Spain, Frequency, *C. perfringens*, *E. coli*, Rotavirus, Coronavirus

## Abstract

**Background:**

Neonatal diarrhoea represents a major disease problem in the early stages of animal production, increasing significantly pre-weaning mortality and piglets weaned below the target weight. Enteric diseases in newborn piglets are often of endemic presentation, but may also occur as outbreaks with high morbidity and mortality. The objective of this study was to assess the frequency of different pathogens involved in cases of recurrent neonatal diarrhoea in Spain.

**Results:**

A total of 327 litters from 109 sow farms located in Spain with neonatal recurrent diarrhoea were sampled to establish a differential diagnosis against the main enteric pathogens in piglets. In total, 105 out of 109 (96.3%) case submissions were positive to one of the examined enteric organisms considered potentially pathogenic (*Escherichia coli*, *Clostridium perfringens* types A and C, *Transmissible gastroenteritis virus* [TGEV], *Porcine epidemic diarrhoea virus* [PEDV] or *Rotavirus A* [RVA]). Fifty-eight out of 109 (53.2%) submissions were positive for only one of these pathogens, 47 out of 109 (43.1%) were positive for more than one pathogen and, finally, 4 out of 109 (3.7%) were negative for all these agents. *Escherichia coli* strains were isolated from all submissions tested, but only 11 of them were classified into defined pathotypes. *Clostridium perfringens* type A was detected in 98 submissions (89.9%) and no *C. perfringens* type C was found. Regarding viruses, 47 (43.1%) submissions were positive for RVA, 4 (3.7%) for PEDV and none of them for TGEV.

**Conclusion:**

In conclusion, *C. perfringens* type A, *E. coli* and RVA were the main pathogens found in faeces of neonatal diarrheic piglets in Spain.

## Background

Neonatal diarrhoea is one of the most frequent clinical signs in young piglets, increasing significantly pre-weaning mortality and the number of piglets weaned below the target weight [1]. Enteric diseases in newborn piglets are often endemic, but may also occur as outbreaks with high morbidity and mortality [2, 3]. Although the latter ones have a major economic impact in the short-term, the economic consequences of endemic and recurrent neonatal diarrhoea can also be substantial. In studies conducted in Sweden and Denmark, diarrhoea was estimated to account for 5–24% of the overall pre-weaning mortality [4] and for a reduction in average daily gain (ADG) by 8–14 g/day in the first week of life [5, 6]. Based on these production losses, the cost of neonatal diarrhoea was recently estimated to be 134 € per sow per year in affected herds [7].

Diarrhoea can be described as faeces with excess of water in relation to faecal dry matter [8]. Clinical signs and impact of enteric diseases in newborn piglets vary depending on the infectious agent/s involved, as well as the susceptibility of piglets to infection [9]. Regardless of the cause, a rapid loss of water, electrolytes and nutrients do occur. Given the limited body reserves of the newborn piglet, this situation quickly leads to a deleterious condition of the piglet that may die in a matter of hours. Therefore, acute diarrhoea in the newborn pig should always receive immediate attention [10].

Diarrhoea is a clinical sign and its treatment depends on the nature of the cause; therefore, a definitive diagnosis is required to take the proper control approach. Once management problems are solved, the most common causes of enteric problems are infections by one or more pathogens or changes in the microbiota [9]. The differential diagnosis includes viruses, bacteria and parasites. Among infectious causes of neonatal diarrhoea (first week of age), both bacteria and viruses may play a significant role, not only by separate but also in co-infection [9, 11].

The most widespread viral infection causing diarrhoea in neonatal swine is *Rotavirus type A* (RVA), although types B and C are also linked to enteric diseases as well [12–14]. RVA is often detected as the sole infectious agent, but the majority of studies are based on qualitative detection of RVA without histologic verification; this fact implies that the causative significance of this virus is often unknown. *Transmissible gastroenteritis* (TGE) *virus* (TGEV) and *Porcine epidemic diarrhoea* (PED) *virus* (PEDV) can also cause diarrhoea in piglets [15]. TGEV is a cause of disease in most pig-producing areas of the world [16], but the clinical impact of TGEV in Europe is low since 1980s–90s due to the extensive spread of the closely related *Porcine respiratory coronavirus* (PCRV), which resulted in immunological cross-protection [16]. PEDV was recognized for the first time in Europe during the seventies, but a major concern re-appeared in the European swine production in 2014 when the virus was detected in several countries causing high piglet mortality and significant economic losses [17]. Outbreaks of PED in suckling piglets are clinically indistinguishable from outbreaks of TGE [18].

A number of bacteria are related to neonatal diarrhoea in piglets*. Escherichia coli* is probably the most important one, responsible for a range of different diseases in both man and animals [19]. The main pathotype of *E. coli* responsible for intestinal disease in pigs is enterotoxigenic *E. coli* (ETEC) [20]. However, there are other *E. coli* pathotypes also linked with piglet disease, such as enteropathogenic *E. coli (*EPEC) and those known as attaching-and-effacing (A/E) *E. coli* [21]. *Clostridium perfringens* is another important diarrhoeagenic pathogen, which is classified into five groups (A to E) based on the production of the major toxin types alpha, beta, epsilon and iota [22]. *C. perfringens* type C produces α- and β-toxin and can cause high mortality rates in newborns [23]. *C. perfringens* type A produces α-toxin and ß2-toxin; besides being considered a cause of neonatal diarrhoea, it is also considered part of the normal intestinal microbiota of piglets [24].

The objective of the present work was to investigate the presence of viral (RVA, TGEV and PEDV) and bacterial (different *E. coli* pathotypes, and *C. perfringens* types A and C) pathogens associated to cases of recurrent neonatal diarrhoea in Spain.

## Methods

### Case selection

A total of 327 litters from 109 sow farms located in Spain that experienced neonatal recurrent diarrhoea were sampled to establish a differential diagnosis against enteric pathogens in piglets. Therefore, a convenience sampling was designed; the inclusion criterion for all veterinarians submitting samples was the presence of diarrhoea during the first week of age in a repeated fashion through different batches.

Five piglets per litter from three litters with diarrhoea were sampled in each studied farm. Selected piglets were not treated with antibiotics and their faeces were collected individually and pooled subsequently in a plastic tube (total of 3 pools per farm, one per each litter). A swab in Amies medium was taken from each plastic tube, which was previously hand-shaken. Both samples, swab and plastic tube, were sent to IVD Innovative Veterinary Diagnostics (IVD) Laboratory (Germany) to check for several enteric pathogens causing neonatal diarrhoea. The shipment was sent by plane, with a delivery in 24 h and using a cool pack in the box during the transportation.

### Bacteriological isolation

Faeces from the swab were streaked out on blood agar (Columbia agar with sheep blood; Oxoid: PB5008A) and Gassner agar (Oxoid: PO5021A) for the isolation of *Escherichia coli*. Inoculated plates were incubated aerobically at 37 °C for 24 h. Suspected *E. coli* isolated colonies were confirmed by morphological characteristics; in addition, blue colonies (lactose-positive) on Gassner agar were further confirmed by negative oxidase testing. Haemolysin presence was identified by haemolysis on blood agar.

For the isolation of *C. perfringens*, faeces from the swab were streaked out on plates containing Schaedler agar with sheep blood (Oxoid: PB5034A) and incubated anaerobically (AnaeroGen, Oxoid: AN0035A) at 37 °C for 24 to 48 h. *C. perfringens* was identified because of the double-zoned haemolysis, typical colony morphology and butyric acid smell. Suspected isolated colonies were further confirmed by PCR genotyping using the species-specific cpa-gene coding for the α-toxin.

### *Escherichia coli* Genotyping

*E. coli* species were characterized by the detection of the *E. coli* species-specific glyceraldehyde phosphate dehydrogenase gene gapA [25], which was the internal amplification and positive control in the multiplex PCR used for genotyping. For genotyping purposes, a single *E. coli* colony from a pure agar culture was selected and suspended in 150 μL lysis buffer (10 mM Tris-HCl (pH 8.0), 0.05% Tween 20, 240 μg/mL Proteinase K with 0.5% Triton X-100, and then incubated for 60 min at 60 °C. Subsequently, the suspension was heated at 97 °C for 15 min and the lysate was cooled at room temperature. A total of 1.5 μL of this lysate were used as a template for genotyping PCR methods. Virulence associated genes listed in Table 1 were investigated for *E. coli* isolates by multiplex PCRs [26]. Pathotypes were defined following the combination of detected genes [21].

**Table 1:** Target genes of *Escherichia coli* virulence factors detected by multiplex PCRs

Bacterial components or products	Target genes	Virulence factors
Fimbriae	*faeG (F4)*	F4 fimbriae
	*fanC (F5)*	F5 fimbriae
	*fasA (F6)*	F6 fimbriae
	*fedA (F18)*	F18 fimbriae
	*fim41A (F41)*	F41 fimbriae
	*fimH (F1)*	type 1 fimbriae
	*fimA (F1)*	type 1 fimbriae
Adhesins	*papC*	P fimbriae
	*aidA (AIDA)*	AIDA-I autotransporter adhesin
	*paa*	porcine attaching-effacing associated protein
	*eaeA (intimin)*	intimin
Toxins	*eltB (LTI)*	heat-sensitive enterotoxin
	*estA (STI)*	heat-resistant enterotoxin
	*estB (STII)*	heat-resistant enterotoxin
	*astA (EAST)*	heat-resistant enterotoxin
	*stx2e*	Shiga toxin variant 2e
	*cdtB*	cytolethal distending toxin
	*cnf1*	cytotoxic necrotizing factor type 1
Others	*iucD*	aerobactin siderophore
	*escV*	type III secretion system
	*pic*	serin protease autotransporter

### *Clostridium perfringens* Immunoblotting and genotyping

For the immunoblotting, 5 colonies were suspended in 5 mL TYCG-medium plus TYCG-supplement (liquid media for *Clostridium* spp.) and incubated 6 to 8 h at 37 °C under anaerobic conditions; 1 mL was centrifuged at 11.000 x g for 5 min. The supernatant was used to detect α-, β- and β2-toxins by immunoblotting [27]; α and β toxin presence was defined by positive or negative, and only β2 production was semi quantitatively recorded (strongly positive, positive, weakly positive and negative) [28]. For genotyping purposes, a single colony from a pure agar culture was selected and suspended in 150 μL lysis buffer, incubated for 90 min at 60 °C and then heated at 97 °C for 15 min. Lysate cooled at room temperature and genotyping (cpa, cpb, and cpb2 genes) was performed following a previously published method [28].

### Virological analyses

Nucleic acid was extracted from faecal samples using an RNA-DNA isolation kit (MagMAX Pathogen RNA/DNA Kit; Life Technologies GmbH, Darmstadt, Germany). Briefly, 0.5 g of faeces was suspended in 1 mL PBS and vortexed vigorously for 3 min, until the solution was fully suspended. For a separation of phases, it was centrifuged at 100 x g for 3 to 5 s. A total of 200 μL of the solution was transferred to a tube with 500 μL prepared lysis/binding solution of the RNA-DNA isolation kit. It was vortexed vigorously for 5 min and then centrifuged at 16.000 x g for 3 min to clarify the lysate. Nucleic acid isolation was performed with an automated nucleic acid isolation processor (MagMAX™ Express-96 Magnetic Particle Processor; Life Technologies GmbH) based on magnetic bead technology, according the manufacturer’s protocol and instructions. Extracted nucleic acid was analysed by PCR methodologies.

RVA RNA was detected by a previously described real-time RT-PCR [29]. PEDV and TGEV were detected by a real-time multiplex RT-PCR for the simultaneously detection of both viruses, following manufacturer recommendations (virotype PEDV/TGEV RT-PCR Kit, Qiagen, Denmark).

## Results

Globally, 105 out of 109 (96.3%) herds were positive to at least one of the examined enteric pathogens (pathogenic *E. coli*, *C. perfringens* types A and C, TGEV, PEDV or RVA). Fifty-eight out of 109 (53.2%) submissions were positive for only one of these pathogens, 47 out of 109 (43.1%) were positive for more than one pathogen and, finally, 4 out of 109 (3.7%) were negative for all these agents.

*E. coli* strains were isolated from all submissions tested. However, only 11 of them were classified into defined pathotypes (ETEC and EPEC). Specifically, 9 isolates were classified as ETEC and 2 as EPEC (Table 2). Within ETEC isolates, 7 different virulence factor profiles were found; among ETEC strains, the most frequently detected virulence factors were STII (7 isolates), STI (7 isolates) and F4 (5 isolates). Both EPEC strains showed the same combination of virulence factors, Intimin and escV.

**Table 2:** Patothypes of the 11 *E. coli* strains isolated in the present study and their combinations of virulence factors detected by PCR

Pathotype	Combination of virulence factors	Number of isolates
ETEC	F4, STI, STII	2
	AIDA, STII	2
	F4, LTI, STI, STII	1
	F5, intimin, STI	1
	F18, paa, STI, STII	1
	F4, AIDA, LTI, STI, STII	1
	F4, LTI, STI	1
EPEC	Intimin, escV	2

ETEC enterotoxigenic, *E. coli* EPEC enteropathogenic *E. coli*

*C. perfringens* type A was isolated from 98 submissions (89.9%), which were all of them cpa positive by PCR. No isolation of *C. perfringens* type C was found, and all submissions resulted negative to cpb gene detection by PCR. The cpb2 toxin gene was found in 95 out of 98 (96.9%) *C. perfringens* type A isolates. By immunoblotting of these 95 strains, 20 isolates were β2 toxin strongly positive, 51 positive, 17 weakly positive and 7 negative. The result of production of α toxin by immunoblotting was positive in 34 isolates.

Regarding viruses, 47 out of 109 (43.1%) submissions were positive for RVA, 4 (3.7%) for PEDV and none of them for TGEV.

Table 3 summarizes all combinations of pathogens found in the studied diarrhoea cases. Noteworthy, pathogenic *E. coli* were only found in combination with other pathogens, and the maximum number of pathogens found in one submission was four (ETEC, *C. perfringens* type A, RVA and PEDV).

**Table 3:** Combinations of different pathogens found in neonatal cases of diarrhoea in Spain

Combination of pathogens	Number of cases (percentage)
RVA and *C. perfringens* type A	34 (31.2%)
ETEC, RVA and *C. perfringens* type A	4 (3.7%)
PEDV and *C. perfringens* type A	2 (1.8%)
ETEC and *C. perfringens* type A	3 (2.7%)
ETEC, RVA, *C. perfringens* type A and PEDV	1 (0.92%)
ETEC, *C. perfringens* type A and PEDV	1(0.92%)
EPEC, RVA and *C. perfringens* type A	1 (0.92%)
EPEC and *C. perfringens* type A	1 (0.92%)
Total	47 (43.1%)

*PEDV* Porcine epidemic diarrhoea virus, *RVA* Rotavirus type A, ETEC enterotoxigenic *E. coli*, EPEC enteropathogenic *E. coli*

## Discussion

The emergence of the modern pig production, with more intensified farming, has been paralleled with an increase of neonatal piglet diarrhoea prevalence [20]. Neonatal enteric problems in a herd are usually the result from the interaction of multiple factors that need to be examined to find rational means for intervention. Major determinants for the manifestation of neonatal diarrhoea include factors such as passive immunity transferred by colostrum and milk [30], environmental temperature [31] and humidity [21], management [32] and infection pressure by specific pathogens of the herd [32]. The present study focused on different enteric pathogens able to cause neonatal diarrhoea in piglets in an important European pig producing country such as Spain, with specific focus on frequency of infections and co-infections. It was not possible, however, to address the specific role of other non-tested pathogens as well as non-infectious factors in the studied cases.

Both viral and/or bacterial pathogens were found in 96% of the submissions, which represents a fairly high number of cases with at least one infectious agent present. *C. perfringens* type A is considered rather an ubiquitous bacteria in the pig intestinal tract [24]. Therefore, it is difficult to predict if it acted as primary pathogen or its multiplication was triggered by other infectious or non-infectious factors. In consequence, it was not possible to assess in which number of submissions the primary cause was a pathogen. Noteworthy, infectious diarrhoea in newborn piglets is usually related to the presence of a single pathogen and mixed infections are considered less common [33]. However, almost half of the cases (43.1%) of the present study corresponded to mixed infections, with *C. perfringens* type A being present in all these cases.

Regarding viruses, RVA was the most frequently detected agent. Rotavirus is among the most prevalent pathogens in cases of neonatal diarrhoea, and is often detected as the sole infectious agent [34]. In fact, several recent studies have described such high prevalence in pigs in Europe and North America [13; 14]. In contrast, only 4 submissions were positive to PEDV. After the 1980s, problems with PEDV in Europe declined and disease outbreaks have been occasionally seen during the last decades [17]. In 2013, PEDV was introduced for the first time on the American continent and resulted in severe outbreaks of disease in the naïve population [17]. Recently, PEDV isolates similar to the S-INDEL variants described in America have also been detected in Europe [35, 36]. Regarding the occurrence of PED in 2014–2015 recorded by the European Food Safety Authority (EFSA) Network, countries voluntarily reported 245 cases of pig herds meeting the PED case definition and 71 pig herds with RT-PCR confirmation of PEDV-genome; such PEDV-confirmed cases were found in Austria, Belgium, Spain, France, Italy, the Netherlands and Germany (EFSA, 2015). As expected, TGEV was not found in any of the submissions. TGEV is a cause of disease in most pig-producing areas of the world [16]. However, outbreaks of TGE in Europe are rare, probably due to immunological cross-protection induced by PRCV, which is apparently ubiquitous in the continent [16].

ETEC has commonly been incriminated as the main aetiological agent of neonatal diarrhoea [37], although a recent survey conducted in Canada has suggested that the clinical importance of neonatal *E. coli* enteric problems have decreased during recent years [34]. Pathogenic pathotypes of *E. coli* were found only in 10% of the submissions in the present study which may reflect such a decrease in prevalence. Anyway, it is known that the major pathotype of *E. coli* responsible for intestinal disease in pigs is ETEC [20] and these data fit with the results of the current work, since 9 out of 11 *E. coli* pathotypes were ETEC (with major virulence factors being STII and STI toxins and F4 fimbriae). Curiously, two of the *E. coli* isolates consisted of EPEC strains, which are usually related with post-weaning diarrhoea [21]. However, EPEC isolates from neonatal diarrhoea have been already described [38, 39]. In contrast, all tested submissions yielded non-pathogenic *E. coli* strains, which is in line with other published works. In a Danish study on neonatal diarrhoea in four commercial swine herds, non-haemolytic *E. coli* were the most predominant isolate obtained after aerobic culturing of both diarrhoeic and non-diarrhoeic piglets [40]. This was also the case in a similar Swedish study on neonatal diarrhoea, where non-haemolytic *E. coli* was found in all piglets [41]. The prevalence of classical porcine ETEC in both Scandinavian studies was very low in diarrhoeic piglets (less than 3%). This fact can be associated to modern swine production, in which pre-farrowing vaccination of sows against *E. coli* fimbriae antigens (F4, F5 and F6) is common.

*C. perfringens* type A was found in most of the submissions (89.9%), with detection of the cpb2 gene by PCR in the majority of cases. Moreover, in 88 cases such results were reinforced with the production in vitro of the β2 toxin by immunoblotting. This result fits well with a recent report from Poland [42], who found a 91.4% prevalence of *C. perfringens* type A at herd level. Such a high rate of detection raises the question of this bacterium as a true cause of diarrhoea or part of the microbiota, which may be up-regulated in enteric disease scenarios. In fact, the impact of α and β2 toxins on disease pathogenesis has not been conclusively answered [43]. In contrast, *C. perfringens* type C was not detected in any of the samples. This bacterium causes disease in piglets in many areas of the world, but in a global perspective, it is considered much less important than other enteric pathogens [22]. In the same Polish survey, for example, they found a 1.4% herd prevalence of this bacterium [42]. Probably, during recent years, *C. perfringens* type C infections are rare in cases of neonatal diarrhoea due to sow vaccination [34]. Indeed, in Spain, routine vaccination of sows pre-farrowing with beta toxoid vaccines is usual.

## Conclusions

In summary, the present study shows that RVA, ETEC and *C. perfringens* type A are the main pathogens involved in persistent neonatal diarrhoea in Spain. In almost half of the cases, more than one enteric pathogen was found. Noteworthy, pathogenic *E. coli* was only found in combination with other pathogens, and the maximum number of pathogens found in one submission, occurring once, was four.
